# Amoeba-Resisting Bacteria and Ventilator-Associated Pneumonia

**DOI:** 10.3201/eid0907.030065

**Published:** 2003-07

**Authors:** Bernard La Scola, Ioanna Boyadjiev, Gilbert Greub, Atieh Khamis, Claude Martin, Didier Raoult

**Affiliations:** * Unité des Rickettsies, Marseille, France; † Hôpital Nord, Marseille, France

**Keywords:** Pneumonia, Ventilator-associated pneumonia, Legionella, Bosea, Legionnaire’s disease, amoeba, research

## Abstract

To evaluate the role of amoeba-associated bacteria as agents of ventilator-associated pneumonia (VAP), we tested the water from an intensive care unit (ICU) every week for 6 months for such bacteria isolates; serum samples and bronchoalveolar lavage samples (BAL) were also obtained from 30 ICU patients. BAL samples were examined for amoeba-associated bacteria DNA by suicide-polymerase chain reaction, and serum samples were tested against ICU amoeba-associated bacteria. A total of 310 amoeba-associated bacteria from10 species were isolated. Twelve of 30 serum samples seroconverted to one amoeba-associated bacterium isolated in the ICU, mainly *Legionella anisa* and *Bosea massiliensis,* the most common isolates from water (p=0.021). Amoeba-associated bacteria DNA was detected in BAL samples from two patients whose samples later seroconverted. Seroconversion was significantly associated with VAP and systemic inflammatory response syndrome, especially in patients for whom no etiologic agent was found by usual microbiologic investigations. Amoeba-associated bacteria might be a cause of VAP in ICUs, especially when microbiologic investigations are negative.

Hospital-acquired pneumonia occurs in 0.5% to 1% of admitted patients admitted, representing 10% to 15% of all nosocomial infections; pneumonia is the most common cause of nosocomial infection in intensive-care units (ICUs) (*1*). This pneumonia is associated with high death rates. As the etiologic agent of pneumonia remains unknown in 20% to 50% of cases (*2*), identifying new lung pathogens is a major public health goal. Aquatic bacteria such as *Legionella* spp., *Pseudomonas* spp.*, Stenotrophomonas* spp., *Burkholderia* spp., or *Acinetobacter* spp. may colonize in hospital water supplies and have previously been shown to be causally associated with cases of nosocomial infections (*3*). Free-living amoebae have been shown to be a reservoir of pathogens such as *Legionella* sp., *Burkholderia picketti,* and *Cryptococcus neoformans* (*4*–*7*). The most studied amoebae-associated bacterium is *Legionella pneumophila,* the agent of Legionnaires’ disease (*8*), which frequently results from exposure to contaminated aerosols. Additional amoeba-associated bacteria might be implicated in community-acquired pneumonia, including *Legionella*-like amoebal pathogens (*9*) and members of the genus *Parachlamydia* (*10*). As part of research into the diversity of bacterial agents associated with amoebae in hospital water supplies, we have identified a new α-Proteobacteria belonging to the *Bradyrhizobiaceae* family (*11*–*13*). We demonstrated that patients with nosocomial pneumonia hospitalized in the vicinity of the contaminated water in a public hospital of our city have elevated antibody titers to these bacteria (*14*). In this study, we performed the same kind of analysis but focused our work on a single ICU during a 6-month period. Amoeba-associated bacteria were periodically evaluated in all ICU water taps. To evaluate contact of patients hospitalized in this ICU and amoeba-associated bacteria in the water, serum and bronchoalveolar lavages (BAL) samples were periodically sampled. Serum samples were tested in an immunofluorescence assay against the isolated bacteria to detect seroconversions, and DNA of these bacteria were detected in BAL samples by suicide-polymerase chain reaction (PCR) (*15*,*16*), a PCR technique without positive controls that incorporates “disposable” primers to avoid false-positive results. The second part of this work was to evaluate if exposure to the amoeba-associated bacteria in the ICU could be associated with disease. Thus, we specifically studied some clinical markers of infection, including fever, systemic inflammatory response syndrome (SIRS), and pneumonia for patients admitted to the ICU. As a definition of pneumonia based only on clinical and roentgenographic criteria has been criticized for low specificity (*17*–*19*), we used strict criteria in the definition. These criteria were applied to cases in which bacterial documentation was negative to determine if disease observed in patients hospitalized in an ICU may be attributed to amoeba-associated bacteria.

## Materials and Methods

All patients admitted to the ICU during a 26-week period who needed intubation and mechanical ventilation were included. Patients were evaluated at admission by the Acute Physiology and Chronic Health Evaluation II score (*20*). At admission and every week thereafter, temperature, leukocytes and platelet counts, hepatic enzymes, and presence of SIRS and ventilator-associated pneumonia (VAP) were recorded. Serum samples were obtained from patients at admission, every 7 days afterwards, and at discharge. BAL was obtained at admission immediately after intubation by using protected mini-bronchoalveolar lavage (Combicath, Plastimed, Le-Plessy-Bouchard, France), then performed when a lung infiltrate suggestive of pneumonia appeared, and repeated every week until pneumonia resolved. BALs are part of the routine diagnosis and follow-up of pneumonia in the ICU and were not performed specifically for the study. Informed consent was obtained from the patient’s family, according to French legislation. Data about bacteria isolated from blood cultures, lung secretions, and urine by conventional procedures were recorded. When isolated from urine and lung secretions, bacteria were only considered as pathogenic when concentration in the specimen was >10^5^ and >10^6^ CFU/mL, respectively (*21*).

Definitions of VAP and SIRS were based on previously published criteria (*22*–*25*), but to increase specificity, we limited our study to severe cases of VAP by adding strict criteria (Table 1). Taps and ice machine water were sampled every other week, as previously reported (*11*). The procedure for isolating bacteria from water and lung secretions by using cocultivation with *Acanthamoeba polyphagia* followed by subculture onto BCYE agar plates has been detailed elsewhere (*11*,*26*). Bacteria were identified by using 16S rRNA gene sequence comparisons as previously described (*27*). *Legionella* species were identified by using *mip* gene amplification and sequencing (*28*).

**Table 1:** Definition criteria for ventilator-associated pneumonia (VAP) and systemic inflammatory response syndrome (SIRS)

VAP	SIRS	Unexplained VAP	Unexplained SIRS
New and persistent roentgenographic lung infiltrate and new onset of: a) Increase in white blood cells >10 g/L b) Fever or hypothermia (>38°C or <36°C) c) Purulent sputum d) Duration of at least 2 weeks e) PaO_2_/FiO_2_ ratio <100	At least two of: a) T° >38°C or <36°C b) Heart rate >90/min c) Respiratory rate >20/min or PaCO_2_ <32 mmHg d) Leukocytes >12 or <4 g/L or immature (band) forms	Lack of recovery of bacteria from: a) Lung secretions b) Blood cultures	Lack of recovery of bacteria from: a) Lung secretions b) Blood cultures c) Urine

Twenty bacterial antigens were tested by microimmunofluorescence as previously reported (*14*,*29*). Bacterial species isolated from the ICU water during the studied period and 10 other species previously isolated in the same conditions from other sites (*Bosea eneae, B. vestrisii, B. thiooxydans, Mesorhizobium amorphae, Azorhizobium caulinodans, Afipia felis, A. felis* genospecies A, *A. clevelandensis, A. birgiae,* and *A. massiliae* [*11–14*]) were tested*.* The serologic tests were performed on the first serum samples from all patients admitted to the ICU; these patients were available for a second sample (30 patients). The control group comprised 10 patients in the same ICU. These patients had shorter stays and had samples taken at admission but was not available (100 patients) and 114 patients with other diseases, including Q fever (10 samples), trench fever (5 samples), tularemia (8 samples), Mediterranean spotted fever (10 samples), epidemic typhus (5 samples), syphilis (10 samples), cat scratch disease (5 samples), pneumonia caused by *Chlamydia pneumoniae* (5 samples), *C. psittaci* (5 samples), *Mycoplasma pneumoniae* (10 samples), *L. pneumophila* (10 samples), hepatitis C virus (5 samples), infections caused by cytomegalovirus (5 samples), Epstein Barr virus (11 samples), and HIV (10 samples). Serum samples were diluted at 1:25, 1:50, and 1:100 for immunoglobulin (Ig) G and IgM determination. The cutoff titer for a positive detection was determined as the lowest titer for which all first serum samples and control serum samples were negative. Then, all patients’ serum samples were tested and serial twofold dilutions from 1:50 to 1:1600 were made on these samples with titers at least equal to the cutoff titer. Tests by Western blot were performed as previously described (29) for any patient with a seroconversion.

DNA was extracted from BAL samples by using QIAMP Tissue kit (QIAGEN, Hilden, Germany), according to the manufacturer’s instructions. PCR detection was attempted on all BAL samples for bacteria against which at least one seroconversion was generated. DNA extraction from serum samples was performed in a laboratory other than the one in which the isolates were identified to avoid vertical contamination from previous amplifications, and no positive control was used to avoid horizontal contamination from the same experiment (*16*). We used a nested PCR that incorporated two primer pairs used only once (Table 2) followed by sequencing and comparison to the targeted sequence, as previously described for suicide-PCR (*15*,*16*). All samples were tested the same day in the same assay. Sample testing was blinded, and positive amplicons were sequenced. Negative controls consisted of BAL samples from 10 patients of the same ICU obtained at admission, BAL samples from 200 patients with nosocomial pneumonia hospitalized in other medical centers of the city, water samples, and a suspension of *A. polyphagia.* At least one negative control was used for every two serum samples.

**Table 2:** Polymerase chain reaction primers used for amplification and sequencing of bacterial DNA within human samples

Primers	*Bosea-*related strain	*Legionella anisa*	*Afipia broomeae*	*L. quinlivanii*
External forward	5′-TGCGAGTGTAGAGGTGAAATT-3′	5′-TATTGGTGCTGATTTAGGAA-3′	5′-TCTTTTGTGCGGGAAGATAATG-3′	5′-TTGTTGATGTTTGTTTTGAGACC-3′
External reverse	5′CGCTCGTTGCGGGACTTAA-3′	5′-GCTAAGTCTGAAGGTACA-3′	5′-TAAACTTTCCAACGGCTGGCAT-3′	5′-TTCAACACTTCTTTCATCTGATC-3′
Internal forward	5′-GAGGTGAAATTCGTAGATATT-3′	5′-GCCCAATTGATTTTGACAG-3′	5′-GCTAACTTCGTGCCAGCAG-3′	5′-TCCAAGAATAAAAGGGGATTG-3′
Internal reverse	5′-GAGCTGACGACAGCCAT-3′	5′-GCATTAATTGTAATGCTTCA-3′	5′-GTTTGCTCCCCACGCTTTC-3′	5′-CCATACCATCCTGTAAGCCTT-3′

Comparisons of demographic, clinical, and laboratory data between patients with evidence of amoeba-associated bacteria contact (seroconversion or positive detection in BAL samples) were performed by using chi square and Mann-Whitney tests, respectively. The tested variables were age; sex; an underlying disease; severity score; intubation duration; hospitalization duration; SIRS; death; increase of hepatic enzymes, platelet count, and leukocytes; VAP; and fever. We also compared demographic, clinical, and laboratory data between patients with and without unexplained VAP, fever, and SIRS. Multivariate analysis adjusted for age, sex, prolonged intubation, and an underlying disease was performed to confirm observed associations. STATA software (v. 7.0, Stata Corporation, College Station, TX) was used for analysis.

## Results

Ten species (310 isolates) were identified from 864 water samples (Table 3). *B. massiliensis* and *L. anisa* were the two most commonly isolated species (62.3% of the 310 isolates). In the ICU admission rooms , isolation of *L. anisa* ranged from 75% to 100% of the tested taps during the first 20 weeks; whereas all were negative during the last 6 weeks, after taps were changed. No amoeba-associated bacteria were isolated from BAL.

**Table 3:** Identification of the 310 bacterial strains isolated by using amoebal co-culture procedure

Species	No. of isolates
*Legionella anisa*	126
*Bosea massiliensis*	67
Rasbo bacterium	45
*Bradyrhizobium liaoningense*	29
*L. quinlivanii*	12
*L. pneumophila*	11
*L. rubrilucens*	7
*L. worsleiensis*	6
*B. japonicum*	5
*Afipia broomeae*	2

Ninety serum samples from the 30 patients and the 214 control serum samples were tested by an immunofluorescence assay for IgG and IgM on the 20 amoeba-associated bacteria antigens (12,160 tests). The 30 first serum samples and the samples from blood donors did not have an IgG titer of >1:50 or IgM titer >1:25. Cutoff titers for positive serologic tests with 100% specificity are shown in Table 4. Twelve (40%) patients seroconverted from 10 to 35 days after admission to at least one antigen; 10 showed IgM antibodies (Table 5). Five patients seroconverted to *L. anisa,* six to *B. massiliensis,* including one to both, one to *L. quinlivanii,* and one to *A. broomeae*. Western blots confirmed the seroconversions, with the appearance of several reacting bands on convalescent-phase serum samples (Figure). Patients also seroconverted to amoeba-associated bacteria not detected in the water in this study: two to *A. clevelandensis* (patient 2, IgG=1:100 and IgM=1:100; patient 8, IgG=1:50 and IgM=1:200;) and two to *A. felis* (patient 2, IgG=1:100 and IgM=1:100; patient 9, IgG=1:1600 and IgM=1:25). Seroconversions were significantly more frequent against amoeba-associated bacteria obtained in this ICU than against amoeba-associated bacteria isolated in previous studies: 13 of 300 tests versus 4 of 300 tests and 12 of 30 patients versus 4 of 30 patients (p=0.046 and p=0.039, respectively). Patients also seroconverted more frequently to the most commonly isolated bacteria, *L. anisa* and *B. massiliensis* (>50 isolates, p=0.021).

**Table 4:** Definition of cutoff titers for positive serologic tests and 100% specificity according to antigen tested by using the 224 control serum samples^a^

Antigen	IgG	IgM
*Legionella anisa*	>1:50	>1:25
*Bosea massiliensis*	>1:50	>1:25
Rasbo bacterium	>1:100	>1:25
*Bradyrhizobium liaoningense*	>1:50	>1:25
*L. quinlivanii*	>1:100	>1:25
*L. pneumophila*	>1:50	>1:25
*L. rubrilucens*	>1:200	>1:25
*L. worsleiensis*	>1:200	>1:25
*B. japonicum*	>1:100	>1:25
*Afipia broomeae*	>1:200	>1:25
*Bosea eneae*	>1:800	>1:25
*B. vestrisii*	>1:100	>1:25
*B. thiooxydans*	>1:50	>1:25
*Mesorhizobium amorphae*	>1:100	>1:25
*Azorhizobium caulinodans*	>1:100	>1:25
*Afipia felis*	>1:100	>1:25
*A. felis* genospecies A	>1:100	>1:25
*A. clevelandensis*	>1:100	>1:25
*A. birgiae*	>1:50	>1:25
*A. massiliae*	>1:50	>1:25

^a^Ig, immunoglobulin.

**Table 5:** Antibody titers of 12 serum samples with seroconversion to at least one of the bacteria isolated in the intensive care unit^a^

Case	Wk of sampling	*Bosea massiliensis*		*Legionella anisa*		*L. quinlivanii*		*Afipia broomeae*	
		IgG	IgM	IgG	IgM	IgG	IgM	IgG	IgM
1^a^	1			<1:50	<1:25				
	4			**1:50**	**1:100**				
	7			**1:800**	**1:50**				
2 ^a^	1	<1:50	<1:25						
	3	**1:400**	**1:100**						
7	1			<1:50	<1:25				
	3			<1:50	**1:200**				
8	1					<1:50	<1:25		
	3					**1:200**	**1:800**		
9	1	<1:50	<1:25	<1:50	<1:25				
	3	**1:400**	**1:50**	**1:50**	**1:400**				
	5	**1:50**	**1:25**	**1:800**	**1:50**				
11	1	<1:50	<1:25						
	5	**1:50**	**1:50**						
12	1			<1:50	<1:25				
	3			**1:400**	**1:50**				
13	1							<1:50	<1:25
	3							1:50	<1:25
	5							1:100	<1:25
	7							**1:200**	<1:25
19	1			<1:50	<1:25				
	3			**1:400**	<1:25				
22	1	<1:50	<1:25						
	3	**1:200**	**1:50**						
23	1	<1:50	<1:25						
	5	**1:400**	**1:25**						
28	1	<1:50	<1:25						
	3	**1:400**	**1:25**						
	5	**1:200**	**1:25**						

^a^Patients with positive PCR results in bronchoalveolar lavage samples (patient 1, *L. anisa*; Patient 2, *B. massiliensis*); Ig, immunoglobulin. All patients were sampled at admission (wk 1); titers in bold are those greater than or equal to defined cutoff titers (Table 4).

**Figure:**
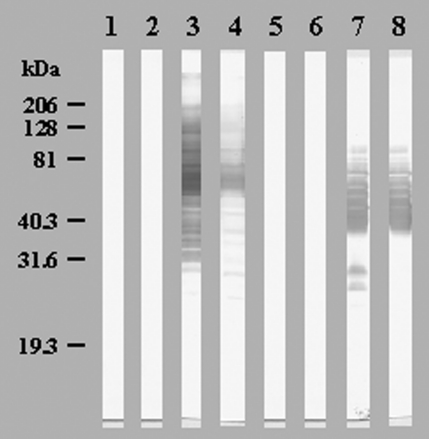
Western blot showing seroconversions in immunoglobulin (Ig) G (Lanes 1, 3, 5, 7) and IgM (Lanes 2, 4, 6, 8) of patient 2 against *Bosea massiliensis* (Lanes 1 to 4) and patient 9 against *Legionella anisa* (Lanes 5 to 8). Lanes 1, 2, 5, 6: acute-phase sera; Lanes 3, 4, 7, 8: convalescent-phase sera.

Table 6 shows the clinical characteristics of patients with serologic evidence of exposure to amoeba-associated bacteria isolated in the ICU. Analysis for risk and potential confounding factors did not indicate differences between patients with or without seroconversion. However, seroconversion was statistically associated with VAP (p=0.026), unexplained VAP (p=0.034), SIRS (p=0.024), and unexplained SIRS (p=0.045). Multivariable logistic regression demonstrated that seroconversion was independently associated with VAP even after we adjusted for intubation duration, hospitalization duration, number of serum samples, and underlying disease (p=0.014 to 0.030).

**Table 6:** Clinical characteristics of patients with or without seroconversion to one of the amoeba-associated bacteria^a^

Clinical characteristics	Seroconversion (N=12)	No seroconversion (N=18)	p value
Demographic data			
Median age in y (IQR)	35 (25–43)	24 (21–52)	0.85
Male (%)	10 (83.3)	15 (83.3)	1
Risk and potential confounding factors			
Underlying disease (%)	2 (16.6)	3 (16.6)	1
Circulation injury (%)	9 (75)	14 (77.8)	1
Median APACHE II^a^ score (IQR)	21 (14–4)	23 (16–34)	0.12
Intubation in ICU (%)	8 (66.6)	7 (38.9)	0.26
Median hospitalization days (IQR)	25 (19–41)	17 (10–23)	0.094
Median intubation duration in days (IQR)	11 (7–20)	11 (7–20)	0.8
Median number of serum samples (IQR)	3 (3–5)	3 (2–4)	0.12
Clinical data			
VAP (%)	10 (83.3)	7 (38.9)	**0.026**
Unexplained VAP (%)	6 (50)	2 (11.1)	**0.034**
Fever > 38.5 °C (%)	12 (100)	17 (84.4)	1
Unexplained fever (%)	7 (58.3)	11 (39.3)	0.27
SIRS (%)	12 (100)	11 (61.1)	**0.024**
Unexplained SIRS (%)	7 (58.3)	3 (14.3)	**0.045**
Death (%)	2 (16.7)	8 (44.4)	0.23
Paraclinical data			
Leukocytes > 12 g/L (%)	12 (100)	14 (77.8)	0.13
Platelets > 500 g/L (%)	5 (41.7)	6 (33.3)	0.7
PCR detection of ARB in BAL samples(%)	2 (17)	0	0.15

^a^IQR, interquartile range; VAP, ventilator-associated pneumonia; SIRS, Systemic Inflammatory Response Syndrome; APACHE II, Acute Physiology and Chronic Health Evaluation II; PCR, polymerase chain reaction; ARB, angiotensin receptor blockers; BAL, bronchoalveolar lavage samples; bold p values are those that are significant (<0.05).

The DNA of *L. anisa* and *B. massiliensis* were each detected once in the 66 BAL samples from 30 patients. For two samples, seroconversion to the identified bacteria, *L. anisa* and *B. massiliensis,* respectively, was observed 4 and 2 weeks after the PCR was positive in BAL. None of the 210 control patients was positive for these bacteria in BAL samples compared to 2 of 30 patients in the ICU (p<0.01).

The death rate was 33.3%; disease was mainly associated with fever (96.6%), VAP (56.6%), and SIRS (76.6%). No microbial etiologic agent was found in 18 (62%) of 29 patients with fever, 10 (43%) of 23 patients with SIRS, and in 8 (47%) of 17 patients with VAP. VAP was significantly associated with duration of hospitalization (median hospitalization days/interquartile range of 23/18–41 with VAP versus 14/10–21 without VAP, p=0.04), SIRS (16/17 with VAP versus 7/13 without VAP, p=0.025) and seroconversion to amoeba-associated bacteria (10/17 with VAP versus 2/13 without VAP, p=0.026). No other statistical difference was observed between patients with VAP and without VAP in terms of demographic, clinical, and paraclinical data, risks, and potential confounding factors. Most patients received cephalothin for the first 3 days of hospitalization as an antibiotic prophylaxis. The most commonly used antibiotics in patients with VAP were third-generation cephalosporins. Patients with VAP received antibiotics more frequently and for more than 1 week (16/17 with VAP versus 7/13 without VAP, p=0.025).

## Discussion

We confirmed that several amoeba-associated bacteria are common in the water in ICU; we recovered 310 amoeba-associated bacteria isolates from 10 species from the water of the ICU. As exposure to a microorganism is a prerequisite to infectious disease, we first evaluated contact of patients hospitalized in the ICU with amoeba-associated bacteria in water. We found that 12 (40%) of 30 of patients seroconverted to amoeba-associated bacteria and that these seroconversions were significantly more common against local isolates than to amoeba-associated bacteria from other ICUs. Moreover, antibody response parallels that of water contamination with *B. massiliensis* and *L. anisa,* which cause 83% of seroconversions identified in 62% of isolates (p=0.021). The cutoff titers, chosen to have 100% specificity, and the detection of several reactive antigens in the Western blots (Figure) suggest that these seroconversions represent specific serologic reactions. Detection of antibodies reacting to amoeba antigens would also be important because infections could occur through inhalation (perhaps after colonization) of infected amoebae acting as a “Trojan horse” (*6*). In future studies, isolation of amoebae in the aquatic environment as in the BAL samples of patients will be performed for further use as antigens.

Patient exposure to amoeba-associated bacteria from the ICU was also evident in results of amoeba-associated bacteria DNA detection in BAL samples from 2 of 30 patients as compared with 0 of 210 control patients (p<0.01). Moreover, for these patients, bacterial DNA was detected in the BAL sample before the seroconversion to the same bacteria 2 to 4 weeks after admission. This rate is compatible with an acute infection occurring during hospitalization rather than colonization of the respiratory tracts of patients. Contamination is unlikely when using a suicide-PCR procedure (*15*,*16*). Isolation of these agents was probably hampered by the antibiotic prophylaxis instituted at admission in this ICU for trauma patients and likely explains the lack of amoeba-associated bacteria isolation in BAL samples.

Among the 30 patients, VAP occurred frequently (56.6%) and was associated with hospitalization duration, as previously reported (*30*,*31*). Of the patients with VAP, 58.8% seroconverted to amoeba-associated bacteria, as compared to 15.4% of the remaining 13 patients (p=0.026). In patients with seroconversion, SIRS was also more prevalent (p=0.024). The percentages of unexplained VAP and unexplained SIRS were four times more common in amoeba-associated bacteria seroconverters than in nonseroconverters and remained statistically significant in spite of the small population studied (Table 6). Thus, amoeba-associated bacteria may be a common cause of unexplained VAP and SIRS.

Finally, we identified a cryptic outbreak in the ICU caused by *L. anisa,* a pathogen commonly encountered in the environment (*32*,*33*), only implicated in a few epidemics of Pontiac fever (*34*,*35*) and four cases of legionellosis (*26*,*36*–*38*). Five (16.7%) of 30 patients were infected with this bacterium, considered a relatively rare pathogen. Serologic tests are not currently used for *L. anisa*, and no urinary antigen test is available. Therefore, diagnosis based only on isolation may explain why *L. anisa* is so rarely reported. However, its absence in the BAL samples from the 200 patients from other ICUs shows that local epidemiology plays a major role. This study confirms that *L. anisa* is common in the environment (*32*,*33*).

Members of the *Bosea* genus are gram-negative, oxidase-positive, catalase-positive rods belonging to the α-2 subgroup of Proteobacteria. All are motile. They grow well on BCYE agar from 25°C to 37°C but do not grow or grow weakly on Columbia agar with 5% sheep blood. Colonies are smooth, mucoid, round, and cream colored and are urease positive and α-hemolytic on Columbia agar with 5% sheep blood and 0.2% yeast extract. *B. massilensis* are negative in assays for arginine dihydrolase activity, esculin and gelatin hydrolysis, β-galactosidase activity, maltose assimilation, and acid production by fermentation or oxidation of substrates tested in API 50 CH (Biomérieux, Marcy l’étoile, France), especially D-glucose, D-fructose, D-mannose, and sucrose. The species of the *Bosea* genus have high MICs to penicillin and amoxicillin and low MICs to doxycycline (*13*). In co-culture with *A. polyphagia, B. vestrisii, B. eneae,* and *B. massiliensis* are phagocyted and form progressively large vacuoles that lead to amoebal lysis; however, they have never been reported as pathogenic agents before. As *B. massiliensis* has not been tentatively isolated elsewhere, whether our findings reflect a local phenomenon or whether the bacterium is widely encountered is not known. We think that our data support the role of *B. massiliensis* in severe VAP, but confirmation is needed to definitely establish a role.

Our study indicates that most patients with VAP received β-lactam agents, mainly amoxicillin-clavulanic acid and third-generation cephalosporin. These antibiotics may have inhibited bacterial culture, which explains why no amoeba-associated bacteria were isolated from BAL samples. *B. massiliensis* has low MICs (<0.5 mg/L) to ceftriaxone, doxycycline, rifampin, and erythromycin (*13*). However, β-lactam agents are also active in vitro on *Legionella* spp., but animal models and clinical studies have demonstrated their inefficacy in the treatment of Legionellosis (*39*).

The results of our study confirm that the bacteriologic tests of hospital water supplies is largely ignored. Our work demonstrates that patients are exposed specifically to the most common water amoeba–associated bacteria in their environment, as evidenced by seroconversion against these bacteria and DNA of these bacteria in BAL samples. The route of infection, even if caused by aerosols generated in the ICU, remains unclear. Patients of this ICU sometime receive water through nasogastric tubes but only bottled, sterile water. However, we cannot exclude mistakes caused by not following recommended procedures. Patients for whom exposure to these bacteria is supported by seroconversion or DNA detection in BAL samples have unexplained VAP and SIRS more commonly. We speculate that amoeba-associated bacteria in the environment of intubated patients may concurrently cause unexplained infections and cryptic outbreaks. Research of new etiologic agents of pneumonia in ICUs should be based on environmental study of each ICU since ecologic findings of amoeba-associated bacteria in water points in hospital vary.

## References

[1] Wenzel RP. Hospital-acquired pneumonia: overview of the current state of the art for prevention and control. Eur J Clin Microbiol Infect Dis. 1989;8:56–60. 10.1007/BF01964121 2495952

[2] Marrie TJ, Durant H, Yates L. Community-acquired pneumonia requiring hospitalization: 5-year prospective study. Rev Infect Dis. 1989;11:586–99. 2772465 10.1093/clinids/11.4.586

[3] Rutala WA, Weber DJ. Water as a reservoir of nosocomial pathogens. Infect Control Hosp Epidemiol. 1997;18:609–16. 10.1086/647684 9309431

[4] Rowbotham TJ. Preliminary report on the pathogenicity of *Legionella pneumophila* for freshwater and soil amoebae. J Clin Pathol. 1980;33:1179–83. 10.1136/jcp.33.12.1179 7451664 PMC1146371

[5] Michel R, Hauröder B. Isolation of an *Acanthamoeba* strain with intracellular *Burkholderia pickettii* infection. Zentralbl Bakteriol. 1997;285:541–57. 9144916 10.1016/s0934-8840(97)80116-8

[6] Barker J, Brown MRW. Trojan horses of the microbial world: protozoa and the survival of bacterial pathogens in the environment. Microbiology. 1994;140:1253–9. 10.1099/00221287-140-6-1253 8081490

[7] Steenbergen JN, Shuman HA, Casadevall A. *Cryptococcus neoformans* interactions with amoebae suggest an explanation for its virulence and intracellular pathogenic strategy in macrophages. Proc Natl Acad Sci U S A. 2001;98:15245–50. 10.1073/pnas.261418798 11742090 PMC65014

[8] Stout JE, Yu VL. Legionellosis. N Engl J Med. 1997;337:682–7. 10.1056/NEJM199709043371006 9278466

[9] Marrie TJ, Raoult D, La Scola B, Birtles RJ, de Carolis E. *Legionella*-like and other amoebal pathogens as agents of community-acquired pneumonia. Emerg Infect Dis. 2001;7:1026–9. 10.3201/eid0706.010619 11747734 PMC2631911

[10] Greub G, Raoult D. *Parachlamydiaceae* potential emerging pathogens. Emerg Infect Dis. 2002;8:625–30. 12023921 10.3201/eid0806.010210 PMC2738484

[11] La Scola B, Barrassi L, Raoult D. Isolation of new fastidious α Proteobacteria and *Afipia felis* from hospital water supplies by direct plating and amoebal co-culture procedures. FEMS Microbiol Ecol. 2000;34:129–37. 10.1016/S0168-6496(00)00084-2 11102690

[12] La Scola B, Mallet MN, Grimont PAD, Raoult D. Description of *Afipia birgiae* sp. nov., *Afipia massiliae* sp. nov. and recognition of *Afipia felis* genospecies A. Int J Syst Evol Microbiol. 2002;52:1773–82. 10.1099/ijs.0.02149-0 12361286

[13] La Scola B, Mallet MN, Grimont PAD, Raoult D. *Bosea eneae* sp. nov., *Bosea massiliensis* sp. nov. and *Bosea vestrisii* sp. nov., isolated from water supplies, and emendation of the genus *Bosea (*Das 1996). Int J Syst Evol Microbiol. 2003. In press. 10.1099/ijs.0.02127-0 12656146

[14] La Scola B, Mezi L, Auffray JP, Berland Y, Raoult D. Intensive care unit patients are exposed to amoeba associated pathogens. Infect Control Hosp Epidemiol. 2002;23:462–5. 10.1086/502086 12186213

[15] Raoult D, Aboudharam G, Crubezy E, Larrouy G, Ludes B, Drancourt M. Molecular identification by “suicide PCR” of *Yersinia pestis* as the agent of Medieval Black Death. Proc Natl Acad Sci U S A. 2000;97:12800–3. 10.1073/pnas.220225197 11058154 PMC18844

[16] Raoult D, Fournier PE, Fenollar F, Jensenius M, Prioe T, de Pina JJ, *Rickettsia africae*, a tick-borne pathogen in travelers to sub-Saharan Africa. N Engl J Med. 2001;344:1504–10. 10.1056/NEJM200105173442003 11357153

[17] A’Court CH, Garrard CS, Crook D, Bowler L, Conlon C, Peto T, Microbiologic lung surveillance in mechanically ventilated patients using non-directed bronchial lavage and quantitative culture. Q J Med. 1993;86:635–48. 8255961 10.1093/qjmed/86.10.635

[18] Kollef MH, Bock KR, Richards RD, Hearns ML. The safety and diagnostic accuracy of minibronchoalveolar lavage in patients with suspected ventilator-associated pneumonia. Ann Intern Med. 1995;122:743–8. 7717596 10.7326/0003-4819-122-10-199505150-00002

[19] Sanchez-Nieto JM, Torres A, Garcia-Cordoba F, El Ebiary M, Carillo A, Ruiz J, Impact of invasive and non-invasive quantitative culture sampling on outcome of ventilator-associated pneumonia: a pilot study. Am J Respir Crit Care Med. 1998;157:371–6. 9476845 10.1164/ajrccm.157.2.97-02039

[20] Knaus WA, Draper EA, Wagner DP, Zimmerman JE. APACHE II : a severity of disease classification system. Crit Care Med. 1985;13:818–29. 10.1097/00003246-198510000-00009 3928249

[21] Reisner BS, Woods GL, Thomson RB Jr, Larone DH, Garcia LS, Shimizu RY. Specimen processing. In: Murray PR, Baron EJ, Pfaller MA, Tenover FC, Yolken RH, editors. Manual of clinical microbiology. 7th ed. Washington: American Society for Microbiology; 1999. p. 64–104.

[22] Kollef MH. Ventilator-associated pneumonia, a multivariate analysis. JAMA. 1993;270:1965–70. 10.1001/jama.270.16.1965 8411554

[23] Salata RA, Lederman MM, Shlaes DM, Jacobs MR, Eckstein E, Tweardy D, Am Rev Respir Dis. 1987;135:426–32. 3101559 10.1164/arrd.1987.135.2.426

[24] American Thoracic Society. Hospital-acquired pneumonia in adults: diagnosis, assessment of severity, initial antimicrobial therapy, and preventive strategies. Am J Respir Crit Care Med. 1996;153:1711–25. 8630626 10.1164/ajrccm.153.5.8630626

[25] Bone RC, Balk RA, Cerra FB, Dellinger RP, Fein AM, Knaus WA, Definitions for sepsis and organ failure and guidelines for the use of innovative therapies in sepsis. Chest. 1992;101:1644–55. 10.1378/chest.101.6.1644 1303622

[26] La Scola B, Mezi L, Weiller PJ, Raoult D. Isolation of *Legionella anisa* using an amoebal coculture procedure. J Clin Microbiol. 2001;39:365–6. 10.1128/JCM.39.1.365-366.2001 11136802 PMC87733

[27] Drancourt M, Bollet C, Carlioz A, Martelin R, Gayral JP, Raoult D. 16S ribosomal DNA sequence analysis of a large collection of environmental and clinical unidentifiable bacterial isolates. J Clin Microbiol. 2000;38:3623–30. 11015374 10.1128/jcm.38.10.3623-3630.2000 PMC87447

[28] Ratcliff RM, Lanser JA, Manning PA, Heuzenroeder MW. Sequence-based classification scheme for the genus *Legionella* targeting the *mip* gene. J Clin Microbiol. 1998;36:1560–7. 9620377 10.1128/jcm.36.6.1560-1567.1998 PMC104877

[29] La Scola B, Rydkina L, Ndihokubwayo JB, Vene S, Raoult D. Serological differentiation of murine typhus and epidemic typhus using cross-adsorption and Western blotting. Clin Diagn Lab Immunol. 2000;7:612–6. 10882661 10.1128/cdli.7.4.612-616.2000 PMC95923

[30] George DL, Falk PS, Wunderink RG, Leeper KVJ, Meduri GU, Steere EL, Epidemiology of ventilator-acquired pneumonia based on protected bronchosopic sampling. Am J Respir Crit Care Med. 1998;158:1839–47. 9847276 10.1164/ajrccm.158.6.9610069

[31] Vincent JL, Bihari DJ, Suter PM, Bruining HA, White J, Nicolas-Chanoin MH, The prevalence of nosocomial infection in intensive care units in Europe. JAMA. 1995;274:639–44. 10.1001/jama.274.8.639 7637145

[32] Gorman GW, Feeley JC, Steigerwalt A, Edelstein PH, Moss CW, Brenner DJ. *Legionella anisa*: a new species of *Legionella* isolated from potable waters and a cooling tower. Appl Environ Microbiol. 1985;49:305–9. 3985609 10.1128/aem.49.2.305-309.1985 PMC238398

[33] Bornstein N, Vieilly C, Marmet D, Surgot M, Fleurette J. Isolation of *Legionella anisa* from a hospital hot water system. Eur J Clin Microbiol. 1985;4:327–30. 10.1007/BF02013661 3894018

[34] Fenstersheib MD, Miller M, Diggins C, Liska S, Detwiler L, Werner SB, Outbreak of Pontiac fever due to *Legionella anisa.* Lancet. 1990;336:35–7. 10.1016/0140-6736(90)91532-F 1973219

[35] Fields BS, Barbaree JM, Sanden GN, Morrill WE. Virulence of *Legionella anisa* strain associated with Pontiac fever: an evaluation using protozoan, cell culture, and guinea pig models. Infect Immun. 1990;58:3139–42. 2117580 10.1128/iai.58.9.3139-3142.1990 PMC313623

[36] Bornstein N, Mercatello A, Marmet D, Surgot M, Deveaux Y, Fleurette J. Pleural infection caused by *Legionella anisa.* J Clin Microbiol. 1989;27:2100–1. 2778073 10.1128/jcm.27.9.2100-2101.1989 PMC267746

[37] Thacker WL, Benson RF, Hawes L, Mayberry WR, Brenner DJ. Characterization of a *Legionella anisa* strain isolated from a patient with pneumonia. J Clin Microbiol. 1990;28:122–3. 2405005 10.1128/jcm.28.1.122-123.1990 PMC269549

[38] Fallon RJ, Stack BH. Legionnaires disease due to *Legionella anisa.* J Infect. 1990;20:227–9. 10.1016/0163-4453(90)91144-3 2341733

[39] Vergis EN, Yu VL. *Legionella* species. In: Yu VL, Merigan TC Jr, Barriere SL, editors. Antimicrobial therapy and vaccines. Baltimore (MD): Williams & Wilkins; 1999. p. 257–72.

